# Changes in Wnt and TGF-β Signaling Mediate the Development of Regorafenib Resistance in Hepatocellular Carcinoma Cell Line HuH7

**DOI:** 10.3389/fcell.2021.639779

**Published:** 2021-08-11

**Authors:** Mustafa Karabicici, Yagmur Azbazdar, Gunes Ozhan, Serif Senturk, Zeynep Firtina Karagonlar, Esra Erdal

**Affiliations:** ^1^Izmir Biomedicine and Genome Center (IBG), Dokuz Eylul University Health Campus, Izmir, Turkey; ^2^Izmir International Biomedicine and Genome Institute (IBG-Izmir), Dokuz Eylul University, Izmir, Turkey; ^3^Genetics and Bioengineering Department, İzmir University of Economics, Izmir, Turkey; ^4^Department of Medical Biology and Genetics, Faculty of Medicine, Dokuz Eylul University, Izmir, Turkey

**Keywords:** hepatocellular carcinoma, regorafenib, Wnt/β-catenin, TGFβ, resistance

## Abstract

Hepatocellular carcinoma (HCC) is an aggressive, chemo resistant neoplasm with poor prognosis and limited treatment options. Exploring activated pathways upon drug treatment can be used to discover more effective anticancer agents to overcome therapy resistance and enhance therapeutic outcomes for patients with advanced HCC. Human tumor-derived cell lines recapitulate HCC diversity and are widely used for studying mechanisms that drive drug resistance in HCC. In this study, we show that regorafenib treatment activates Wnt/β-catenin signaling only in hepatoblast-like HCC cell lines and induces enrichment of markers associated with hepatic stem/progenitor cells. Moreover, activation of Wnt/β-catenin signaling *via* Wnt3a/R-Spo1 treatment protects these cells from regorafenib induced apoptosis. On the other hand, regorafenib resistant cells established by long-term regorafenib treatment demonstrate diminished Wnt/β-catenin signaling activity while TGF-β signaling activity of these cells is significantly enhanced. Regorafenib resistant cells (RRCs) also show increased expression of several mesenchymal genes along with an induction of CD24 and CD133 cancer stem cell markers. Moreover, regorafenib resistant cells also exhibit significantly augmented *in vitro* and *in vivo* migration capacity which could be reversed by TGF-β type 1 receptor (TGFb -R1) inhibition. When combined with regorafenib treatment, TGFβ-R1 inhibition also significantly decreased colony formation ability and augmented cell death in resistant spheroids. Importantly, when we knocked down TGFβ-R1 using a lentiviral plasmid, regorafenib resistant cells entered senescence indicating that this pathway is important for their survival. Treatment of RRCs with TGFβ-R1 inhibitor and regorafenib significantly abolished pSTAT3, pSMAD2 and pERK (44/42) expression suggesting the involvement of both canonical and non-canonical pathways. In conclusion, our data suggest that HCC tumors with aberrant activation in the Wnt/β-catenin pathway, might have higher intrinsic regorafenib resistance and the inhibition of this pathway along with regorafenib administration might increase regorafenib-induced cell death in combinational therapies. However, to resolve acquired regorafenib resistance developed in HCC patients, the combined use of TGF-β pathway inhibitors and Regorafenib constitute a promising approach that can increase regorafenib sensitization and prevent tumor recurrence.

## Graphical Abstract

Wnt and TGF-β pathways mediate the regorafenib resistance.

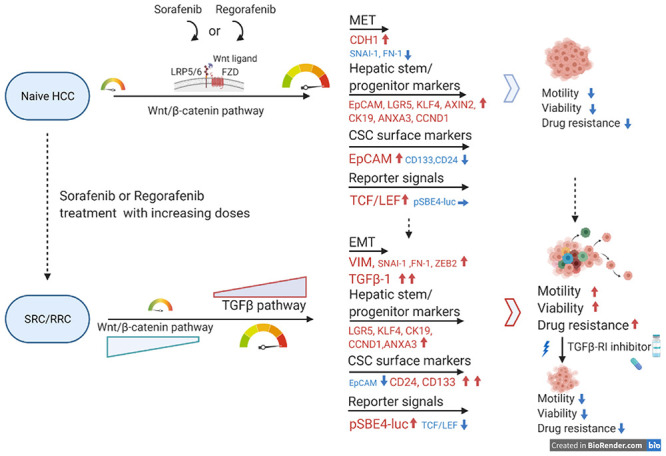

## Introduction

Hepatocellular carcinoma (HCC) is the most common type of liver cancer and the third leading cause of cancer-related deaths worldwide (Bertuccio et al., 2017; Savitha et al., 2017; Yang et al., 2019; Pérez et al., 2020). HCC is an aggressive, chemo resistant neoplasm with complex etiology and high metastatic potential. The majority of diagnosis is done at an advanced stage where HCC patients are not suitable for potentially curative therapies including liver transplantation or surgical resection. Therefore, systemic therapy still is the main therapeutic option for advanced HCC patients (Ohri et al., 2016).

Multi-kinase inhibitors Sorafenib and Lenvatinib, are the two systemic therapies approved first-line for unresectable HCC (Kudo et al., 2018; Xie et al., 2018; Personeni et al., 2019). Moreover, the combination of programmed cell death ligand 1 (PD-L1) inhibitor atezolizumab and VEGFR inhibitor bevacizumab was also recently approved for first-line treatment for advanced HCC. However, since majority of Phase 3 trials are done after sorafenib failure, sorafenib still remains the globally accepted first-line treatment for advanced HCC despite its poor therapeutic response and high rates of resistance (Kudo et al., 2018; Personeni et al., 2019; Kim et al., 2020). Regorafenib, the fluoro analog of sorafenib, is one of the second-line treatments in patients who failed sorafenib therapy. Despite the fact that regorafenib increases the overall survival of patients who progress on sorafenib, the efficacy of this drug is also limited by primary or acquired therapy resistance and high interindividual variability (Bruix et al., 2017; Rimassa et al., 2017; Ettrich and Seufferlein, 2018; Finn et al., 2018; Tovoli et al., 2018). High intra and inter tumor heterogeneity in HCC influences disease progression, prognosis, and variable tumor response of patients to treatment (Ueshima et al., 2017; Iavarone et al., 2019; Hacioglu et al., 2020; Lee M.J. et al., 2020; Lee Y. et al., 2020; Ogasawara et al., 2020). Unfortunately, there are currently no molecular markers for currently used systemic treatments (sorafenib, lenvatinib, regorafenib, and others) in HCC which could be used in therapeutic decision-making to improve patient outcomes. Therefore, it is essential to investigate the molecular mechanisms underlying the variable response to drug treatment and contribute to the development of drug resistance in order to enhance treatment efficacy in HCC.

Human tumor-derived cell lines recapitulate HCC diversity and are widely used for studying mechanisms that drive drug resistance and sensitivity in HCC. Recent characterization of human liver cancer cell lines (LCCLs) identified three subgroups of LCCLs according to the differentiation state and transcriptome (Caruso et al., 2019). The first group of LCCLs include Huh7, HepG2 and Hep3B which express hepato-specific genes and fetal/progenitor markers with active Wnt signaling. Among these cell lines, HepG2 carries a deletion in β-catenin gene (Exon 3–4) resulting in its constitutive activation. In contrast, Hep3B harbors mutations in AXIN1, a major member of β-catenin degradation machinery, leading to stabilization and accumulation of β-catenin protein. Finally, although Huh7 contains wild type β-catenin gene, due to mutations in p53 gene and amplification of FGF19 gene, β-catenin activity is increased in this cell (Cagatay and Ozturk, 2002; Ding et al., 2017).

On the other hand, the other two subgroups of LCCLs are less differentiated with an activation of the TGF-β and noncanonical β-catenin pathways (Caruso et al., 2019; Rebouissou and Nault, 2020). The goal of this study was to analyze the various responses of HCC cell lines to regorafenib treatment and identify molecular pathways that could be used as new potential drug targets for combinational treatment regimens in HCC and/or serve as molecular markers for predicting regorafenib sensitivity.

In this study, we show that regorafenib treatment activates Wnt/β-catenin signaling only in hepatoblast-like HCC cell lines and induces enrichment of markers associated with hepatic stem/progenitor cells. Moreover, Wnt/β-catenin signaling activation by Wnt3a/R-Spo1 treatment protects these cells from regorafenib induced apoptosis. However, regorafenib resistant cells established by long-term regorafenib treatment demonstrate diminished Wnt/β-catenin signaling activity. On the other hand, the TGF-β signaling activity of these cells is significantly enhanced. Regorafenib resistant cells also have increased mesenchymal gene expression along with an induction of CD24 and CD133 cancer stem cell markers. Moreover, TGF-β type1 receptor (TGFβ-R1) inhibition could augment regorafenib induced cell death and reverse increased migration capacity of regorafenib resistant cells. In addition, knocked down of TGFβ-R1 in regorafenib resistant cells induced senescence indicating that this pathway is important for the growth and survival of cells. Treatment of resistant cells with TGFβ-R1 inhibitor and regorafenib significantly abolished pSTAT3, pSMAD2 and pERK (44/42) expression suggesting the involvement of both canonical and non-canonical pathways.

These results suggest that although acute Regorafenib treatment of hepatoblast-like cells initially creates a Wnt/β-catenin pathway-mediated increase in epithelial and stemness related markers, long-term regorafenib treatment of these cells leads to TGF-β pathway activation and the induction of mesenchymal cancer stem cell markers accompanied with an increased *in vivo* metastatic ability mediated by TGF-β pathway. Thus, our data suggest that for HCC tumors with aberrant Wnt/β-catenin activation, the inhibition of this pathway along with regorafenib administration might increase regorafenib-induced cell death and thus ameliorate treatment outcome. However, for acquired regorafenib resistance developed in HCC patients, the combined use of TGF-β pathway inhibitors and Regorafenib constitute a promising approach for regorafenib sensitization and to prevent tumor recurrence.

## Materials and Methods

### Cell Culture

The previously authenticated cell lines were used in this study (Karagonlar et al., 2020). All cells were grown at 37°C, 5% CO2 in RPMI Medium 1640 supplemented with % 2–10 FBS, 1% NEAA, 2 mmol⋅L^–1^ Glutamax, 1% pen/strep. Sorafenib and Regorafenib resistant cells were created by treating parental cell lines with increased doses of drugs starting with their IC50 values. For a period of 8–12 months, at each passage, the drug concentrations were increased between 0.2 and 0.5 μM according to cell viability and proliferation rate of the cells. MTT analysis was performed to confirm the resistance. Established cell lines are maintained under the following drug concentrations: Sorafenib: 7.2 μM and Regorafenib: 8.4 μM.

### Reporter Assays

The plasmids used in TCF/LEF reporter assay were a gift from Dr. Hans Clevers at Hubrecht Institute. Transfections were performed as previously described (Karabicici et al., 2021) using Lipofectamine 2000. For TCF/LEF reporter assays, 24 h after transfection, cells were treated with Regorafenib (5 or 10 μM), Wnt3a/R-Spo1, IWR-1(10 μM) or a combination of these molecules. For TGF-β reporter assays, 24 h after transfection, cells were treated with 5 ng/mL TGF-β1 (Peprotech) (Cat#100-21) or 5 μM TGF-β type 1 receptor (ALK5) inhibitor (Selleckchem) (Cat: #SB525334) for an additional 24 h.

### Fluorescent Staining

Immunofluorescence stainings were performed as previously described (Karabicici et al., 2021), Beta-catenin (BD, 610153)(1:50), p-Beta-catenin (S675)(4176)(1:50), Cytokeratin 19 (sc-6278)(1:50), E-Cadherin (sc8426)(1:50), EpCAM (2929S)(1:50), Vimentin (sc-373717)(1:250), alpha-SMA (ab21027)(1:50), p-Smad2 (S255)(ab188334)(1:50), t-Smad2 (ab40855)(1:50) and Phalloidin-iFluor 488 (ab176753) antibodies were used for the stainings. Cells were visualized using the Confocal LSM 800 microscope.

### Apoptosis Assay

For the detection of apoptosis, cells were stained using AnnexinV Apoptosis Detection Kit (Biolegend; Cat #640922) as described in the manufacturer’s instructions. Annexin V and PI stainings were analyzed using BD LSR Fortessa flow cytometer.

### Real-Time q-PCR

Real-time q-PCR experiments were performed as previously described (Karabicici et al., 2021). using7500 Fast RT PCR System (Applied Biosystems). The relative gene expression was calculated by using the 2^–ΔΔCt^ method. The primers are given in Supplementary Table 1.

### Flow Cytometry

Flow cytometry experiments were performed as previously described (Karabicici et al., 2021). For all cell lines, 2 × 10^6^ cells were seeded into a 10 cm dish 1 day before the experiment. Cells were treated with Regorafenib, Wnt3a/R-spondin or their combination for 24 h and then were stained with EpCAM-FITC (1:50), CD133-APC (1:50) and CD24-APC (1:50) antibodies. Cells were analyzed using the BD LSR Fortessa flow cytometer.

### MTT

3-(4,5-dimethylthiazol-2-yl)-2,5 diphenyltetrazolium bromide (MTT) assay was performed as previously described (Karagonlar et al., 2020). Briefly, cells were seeded in 96-well plates (5 × 10^3^ cell/well) 1 day before the experiment, then treated with increasing concentrations of Sorafenib or Regorafenib (0, 2, 4, 8, 16, 32 μM) for 48 h.

### Colony Formation

For all colony formation assay cells were seeded six well plates at 500–1,000 cells per well. One day later cells treated with 5 μM Regorafenib or 5 μM Sorafenib. The cells were kept in culture for about 7–10 days so that they could form a colony of minimum 50 cells. During this time, fresh medium was added first after 3–4 days and then every 2 days.

At the end of the period, the medium on the cells was removed and cells were washed with 1 × PBS, then fixed with cold methanol for 20 min. After fixation, colonies were stained with crystal violet for 20 min and then washed by immersing them in a container kept under running water to remove excess dye. Plates were dried, then imaged using a camera. Colonies were analyzed with the Fiji cell counter tool of Image J and colony numbers were graphed using GraphPad Prism.

### Spheroid Formation

The hanging drop method was used for the formation of spheroids. Briefly, 1 × 10^3^ cells/30 μl were prepared for all treated or transfected cell lines. Then 30 μl droplets were pipetted on the interior of a 10 cm plate lid. Then the lid was inverted and the plate was incubated for 3 days to start the formation of the spheroids. After 3 days of incubation, plate lids were inverted again and the spheroid medium was collected without disturbing the spheroids. Then the treatment medium was added to the spheroids. After 2 and 4 days in the hanging cell culture, cells were imaged under the stereo microscope using 5× zoom. Then spheroid areas were calculated using threshold calculating methods in the “Adjust” section in ImageJ. Results and statistical analyses were performed with GraphPad Prism.

### Western Blot

Cells were seeded onto 6 cm dishes (1–1.5 × 10^6^ cells/plate) 1 day before treatments. TGFβ-R1 inhibitor (5 μM), Regorafenib (5 μM), Sorafenib (5 μM) or combined treatments were performed for 48 h for all conditions. After treatment, cell media were removed and washed with 1× PBS containing 0.1 mM NaF and 0.1 mM Na3VO4. Then cells were scraped on ice and collected into eppendorf tubes. Cell pellets were lysed using RIPA buffer (150 mM NaCl, %1 NP-40, %0.5 sodium deoxycholate, % 0.1 SDS, 50 mM Tris(pH:8),with 10 mM NaF, 10 mM Na3VO4, 1× phosphatase inhibitor (Pierce,Thermo,A32957,United States) and protease inhibitor (Pierce, Thermo 88666,United States) freshly added. Cell lysates were kept on ice for 30 min and were vortex for every 5 min. Then the cell lysates were sonicated (Diagenode SA Picoruptor 163007 E.C.) for 30 s and centrifuged at max speed for 20 min at 4°C. Supernatants were collected and protein concentrations were calculated using BCA Protein assay kit (Pierce, Thermo, 23225, United States). For all experiments 50 or 100°μg total protein was loaded to jels and incubated overnight with the following antibodies: Akt (p-Ser473)(4060S), Akt (9272),p-Smad3 (Ser423/425)(9520), p-Smad2 (S255)(ab188334),Smad2/3 (D7G7) (8685),Smad4 (sc-7966),p-B catenin (S675)(4176), Beta Catenin (BD, 610153), p-GSK-3α/β (Ser21/9) (9331), GSK-3α/β (sc-7291), p-p38 MAPK (Thr180/Tyr182) (D3F9) (4511),p38 MAPK (9212), p-p44/42 MAPK (Erk1/2) (Thr202/Tyr204) (4370), p44/42 MAPK (Erk1/2) (137F5) (4695), p-Stat3 (Tyr705) (D3A7) (9145),Stat3 (124H6) (9139). Blot images were taken using the Licor (CLX-1137 United States) detection system and analyzed using ImageJ “analyze”-“gel”-“plot lines” tools.

### β-Galactosidase Assay

β-galactosidase assay was performed as previously described (Karabicici et al., 2021). Briefly, cells were seeded on the six well plates (7 × 10^4^) or 12 well plates (2 × 10^4^) 1 day before treatment. Then cells were treated with 5 ng/ml TGF-β1, 5 μM TGFβ -R1 inhibitor or transfected using shTGFβ -R1. 100 nM Doxorubicin treatment for 2 days was used as a positive control. After 6 days, the cell media were removed, cells were washed with 1 × PBS and then were stained with Biovision senescence detection kit as described in the manual. The cells were then imaged using a light microscope and results were analyzed using GraphPad Prism Software.

### Scratch Assay

3–3.5 × 10^5^ cells were seeded on the 12 well plates 1 day before the experiment. Next day, a straight line was drawn using a yellow 200 μl pipette tip from top to down in the center of the cell monolayer. Residual cells were washed and cell media were replaced with control or treatment media. Cells were imaged at day 0, day 1, day 2, and day 3. From the images, wound area was calculated using Image J MRI wound healing tool with a range of 50–100 threshold parameter. Statistical analysis and wound closure percentages were calculated using GraphPad Prism software.

### SubG1 Assay

For the Sub G1 assay all mediums and cells were collected in 50 ml sterile tubes after the treatments (Regorafenib, Wnt3a/R CM (condition medium) or combination) for 48 h. Then the cell pellet was fixed with dropwise addition of cold %70 ethanol to the pellet while vortexing. Cells were kept on ice for 2 h then were centrifuged at 400 × *g* for 5 min. Then the pellet was washed two times with Phosphate-citrate buffer (192 parts of 0.2 M Na2HPO4 and eight parts of 0.1 M Citric acid; pH:7.8). To eliminate RNA, cells were treated with 50 μl 100 μg/ml Ribonuclease A solution for 15 min. After that 450 μl of 50 μg/ml PI was added and cells were incubated for an extra 15 min. Then cells were analyzed at the low flow rate under 400 events/seconds in BD LSR Fortessa flow cytometer and results were analyzed using FlowJo software (Becton Dickinson, Heidelberg, Germany).

### Lentiviral Transfection

Viral plasmids were produced in Hek293T cells as previously described (Karagonlar et al., 2020). 2 × 10^5^ cells/well were plated in six well plates 1 day before the experiment. Next day, cells were transfected with shPLKO.1 empty control plasmid or shTGFB-RI plasmid (TRCN221535, Broad Institute) targeting TGFB-RI transcript. Cells were then with 6 μg/ml puromycin (A1113803, Gibco) for 3 days. After that, puromycin was removed and cells were maintained in their standard growing media.

### Nucleofection

Nucleofection protocol was performed according to Lonza P3 Primary Cell 4D-Nucleofector^TM^ X Kit L. Briefly, 5 μg TCF/LEF 5 μg Renilla and shB-cateninC4 or empty pSUPER plasmids were mixed with 100 μl of nucleofection solution, which contains 5 × 10^5^ cells. Transfection was done using the CA-137 program. Cells were incubated for 10 min in RT after nucleofection and then were seeded in 12 well plates. After 48 h, nucleofection efficiency was measured by Dual-luciferase reporter assay.

### Whole-Mount *in situ* Hybridization

*Tg(7xTCF-Xla.Siam: nlsmCherry)^*ia5*^*(designated *TCFsiam*) zebrafish embryos were crossed with WT embryos. At 8 h postfertilization (hpf), embryos were dechorionated with Pronase enzyme and incubated with 5 μM Regorafenib containing E3 medium for 24 h and 48 h. At the end of the 24 and 48 h incubation times, embryos were fixed in 4% PFA in PBS overnight. mCherry probe synthesis and whole-mount *in situ* hybridizations were performed as described previously (Moro et al., 2012).

### Zebrafish Xenograft

Untreated HuH7 cells (CTRL), HuH7 cells treated with 5 μM Regorafenib (Reg5), Regorafenib resistant cells (RRC), and Sorafenib resistant cells (SRC) were labeled with 2 mg/ml DiI (V2288, Molecular Probes) before injection. The cells were then resuspended to a final density of 40,000 cells/μl in 10%FBS in PBS and injected into the yolk of 2 days old dechorionated embryos (∼250 cells/embryo). The injected embryos were incubated at 34°C in E3 media until 5 dpi.

### Statistics

Statistical analyses were done using GraphPad Prism 7(GraphPad Software, Inc., California, United States) software. Two-tailed unpaired student *t*-test was used to determine statistical significance between 2 experimental groups. Differences between groups were considered as “> 0.05” (n.s.), “≤ 0.05” (^∗^), “≤ 0.01” (^∗∗^), “≤ 0.001” (^∗∗∗^) “≤ 0.0001” (^****^).

## Results

### Regorafenib Increases TCF/LEF Reporter Activity Both *in vitro* and *in vivo*

**FIGURE 1:**
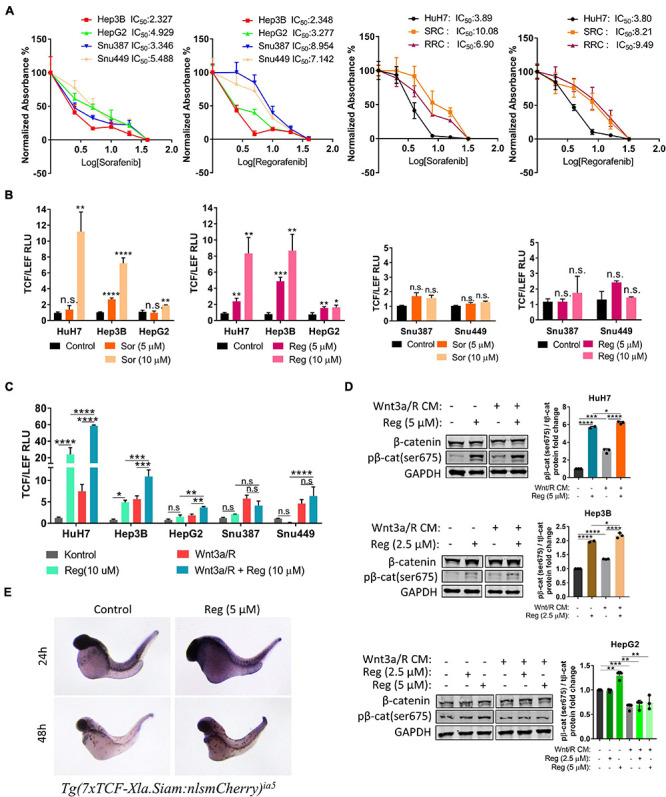
Regorafenib increases TCF/LEF reporter activity both *in vitro* and *in vivo*. **(A)** MTT analysis demonstrates IC50 values for various HCC cell lines. RRC: Regorafenib resistant clone SRC: Sorafenib resistant clone **(B)** TCF/LEF reporter assay shows that sorafenib and regorafenib significantly increase the β-catenin transcriptional activity in hepatoblast-like HCC cell lines but not in mesenchymal-like HCC cell lines. **(C)** Regorafenib enhances Wnt3a/R-Spo1 induced TCF/LEF reporter transcriptional activity in hepatoblast-like HCC cell lines**. (D)** Regorafenib treatment increases phospho-β-catenin in hepatoblast-like HCC cells. **(E)** Whole-mount *in situ* hybridizations using mCherry probe on untreated and regorafenib treated *Tg(7xTCF-Xla.Siam)* Wnt/β-catenin reporter fishes demonstrate that regorafenib treatment increases the TCF/LEF activity *in vivo*. Data represent the average of at least three independent experiments. *p* > 0.05 (n.s.), *p* ≤ 0.05 (*), *p* ≤ 0.01 (**), *p* ≤ 0.001 (***), *p* ≤ 0.0001 (****). Error bars indicate standard deviation (SD).

We first performed MTT analyses to determine IC50 values of drugs on hepatoblast-like (HuH-7, Hep3B, and HepG2), mesenchymal (SNU387, SNU449) cell lines and acquired drug resistant clones of HuH-7 (SRC and RRC). MTT analyses demonstrated that IC50 values of mesenchymal cell lines for regorafenib were significantly higher than hepatoblast-like cells (Figure 1A). Regorafenib treatment also significantly reduced migration, 3-D growth and colony formation of hepatoblast-like cells (Supplementary Figure 1). Of note, we showed that the sorafenib resistant clones also had acquired regorafenib resistance while regorafenib resistant clones had become sorafenib resistant (Figure 1A).

It has previously been shown that hepatoblast-like cell lines exhibit higher basal levels of Wnt/β-catenin pathway activity while mesenchymal-like Snu387 and Snu449 cell lines have repressed Wnt/β-catenin pathway activity (Yuzugullu et al., 2009). So, we investigated if the difference on drug resistance of HCC lines can be explained *via* the characteristics of Wnt/β-catenin signaling. We first analyzed the effect of sorafenib and regorafenib treatment on TCF/LEF reporter activity of HCC cell lines under basal culture conditions as well as with ligand induction. Here we demonstrated that sorafenib and regorafenib treatments alone significantly increase TCF/LEF reporter transcriptional activity of hepatoblast-like cell lines while in mesenchymal-like cell lines Snu387 and Snu449, drug treatments have no significant effect on TCF/LEF reporter activity (Figure 1B). Moreover, although treatment with canonical Wnt pathway ligands Wnt3a/R-Spo1 was able to induce TCF/LEF activity of both hepatoblast like and mesenchymal-like cell lines, additional enhancement of Wnt3a/R-Spo1 induced TCF/LEF reporter activity upon regorafenib treatment was only detected in hepatoblast-like HuH7, HepG2, and Hep3B cell lines (Figure 1C). Notably, regorafenib treatment alone or in combination with Wnt3a/R CM significantly increased TCF/LEF activity and β-catenin phosphorylation in HuH7 cells (Figure 1D).

Moreover, to detect if regorafenib activates Wnt/β-catenin signaling *in vivo*, we utilized *Tg(7xTCF-Xla.Siam)* Wnt/β-catenin reporter fish (Moro et al., 2012). The transgenic fishes were crossed with WT fishes and at 8 hpf, embryos were treated with 5 μM regorafenib for 24 and 48 h. At indicated time points, Wnt/β-catenin reporter activity was detected by *in situ* hybridization. For both time points, TCF/LEF activity was greater in zebrafish treated with regorafenib supporting the activation of Wnt/β-catenin signaling by this drug *in vivo* (Figure 1E).

### Wnt/β-Catenin Activation Protects HuH7 Cells From Regorafenib Induced Apoptosis While Wnt/β-Catenin Inhibition Enhances Cell Death Upon Regorafenib Treatment

**FIGURE 2:**
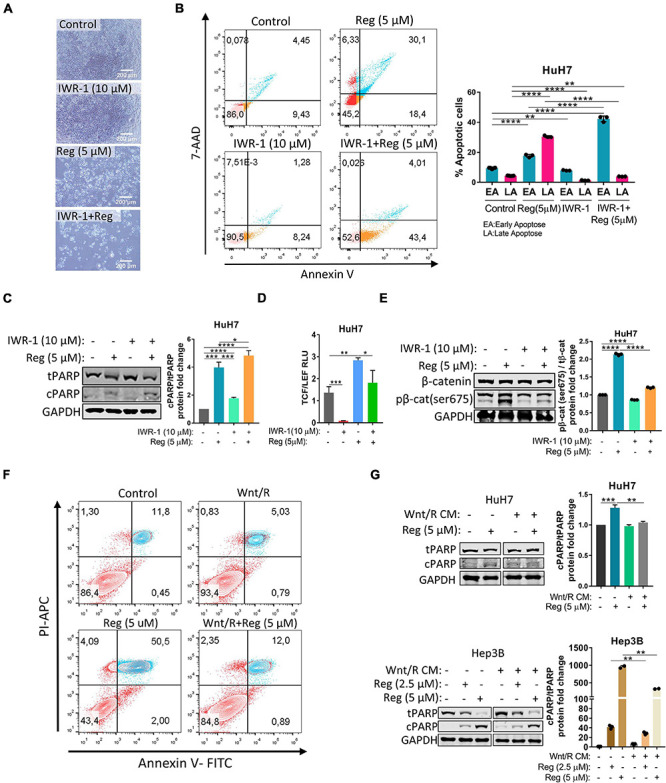
Wnt/β-catenin activation *via* Wnt3a/R-Spo1 treatment protects cells from regorafenib induced apoptosis while Wnt/β-catenin inhibition by IWR-1 augments cell death upon regorafenib treatment. **(A)** Light microscope images indicate augmented cell death upon regorafenib and IWR-1 treatment. Scale bar: 200 μM **(B)** Apoptosis rates of HuH7 cells treated with IWR-1, regorafenib or their combinations were analyzed by Annexin V/PI staining using flow cytometry. **(C)** Western blot analysis of c-PARP and t-PARP in treated cells. **(D)** IWR-1 suppresses basal and regorafenib induced TCF/LEF reporter activity in the HuH7 cell line. **(E)** IWR-1 suppresses basal and regorafenib induced p-β-catenin expression in the HuH7 cell line **(F)** Apoptosis rates of HuH7 cells treated with Wnt3a/R-Spo1, regorafenib or their combinations were analyzed by Annexin V/PI staining using flow cytometry. **(G)** Western blot analysis of c-PARP and t-PARP in treated cells. Data represent the average of at least three independent experiments. *p* > 0.05 (n.s.), *p* ≤ 0.05 (*), *p* ≤ 0.01 (**), *p* ≤ 0.001 (***), *p* ≤ 0.0001 (****). Error bars indicate standard deviation (SD).

To further understand the regulation of the Wnt/β-catenin pathway by regorafenib, we treated HuH7 cells with 10 μM IWR-1 which stabilizes the destruction complex member Axin2 and thus silences the Wnt/β-catenin pathway. When combined with regorafenib, IWR-1 treatment increased Annexin V+/PI- cell population and augmented expression of cleaved PARP (Figures 2A–C and Supplementary Figure 2). Moreover, IWR-1 treatment significantly decreased basal and regorafenib-induced TCF/LEF reporter activity (Figure 2D) and β-catenin phosphorylation (Figure 2E) in the HuH7 cell line. On the other hand, when regorafenib was used in combination with Wnt3a/R-Spo1, regorafenib-induced cell death was greatly reduced (Figure 2F). We also detected a significant decrease in cleaved PARP levels in Wnt3a/R-Spo1treated cells (Figure 2G) and a decrease in sub-G1 cell population (Supplementary Figure 2). Taken together, these findings indicate that Wnt/β-catenin signaling activation prevents regorafenib-induced apoptosis while its inhibition can enhance cell death upon regorafenib treatment.

### Regorafenib Treatment Induces Epithelial and Stemness-Related Gene Expression

**FIGURE 3:**
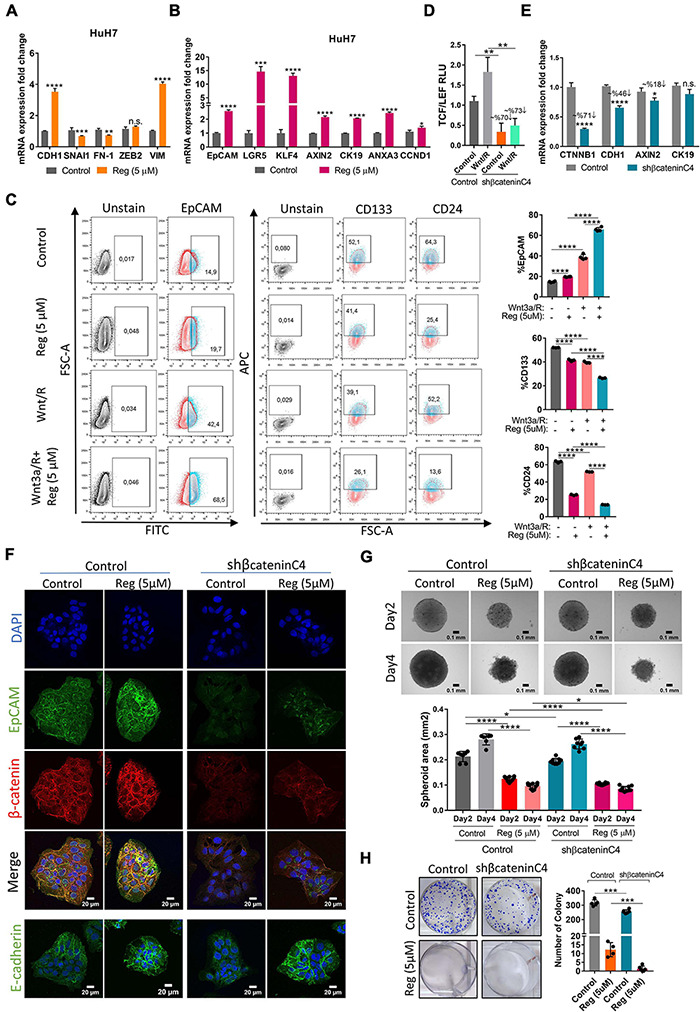
Regorafenib treatment induces epithelial and stemness related gene expression. **(A)** Expression of mesenchymal and epithelial genes as well as **(B)** hepatic stem/progenitor genes were analyzed by q-PCR. Data represent the average of at least three independent experiments. **(C)** Cancer-stem cell marker (EpCAM, CD133 and CD24) positive cell populations were analyzed *via* BD LSR Fortessa flow cytometer with or without Wnt3a/R-Spo1 and regorafenib treatments. **(D)** TCF/LEF reporter activity in Wnt3a/R-Spo1 treated and untreated control and β-catenin knocked down cells **(E)** Expression of Wnt/β-catenin target genes were analyzed by q-PCR. **(F)** Expressions of EpCAM, E-CAD and β-catenin were analyzed in control and β-catenin knocked down cells by immunofluorescence. Images were acquired on a Carl Zeiss LSM 880 AxioObserver confocal microscope with a C-Apochromat 40x/1.2 W, Korr FCS M27 objective. **(G)** 3D spheroids were formed from control and β-catenin knocked down cells with or without regorafenib treatment. Spheroid area was calculated using Image J. **(H)** Colony formation assay was performed using control and β-catenin knocked down cells with or without regorafenib treatment. *p* > 0.05 (n.s.), *p* ≤ 0.05 (*), *p* ≤ 0.01 (**), *p* ≤ 0.001 (***), *p* ≤ 0.0001 (****). Error bars indicate standard deviation (SD).

To evaluate the effect of regorafenib treatment on EMT/MET transition, which is one of the essential hallmarks of cancer progression and metastasis, we detected the expression of epithelial and mesenchymal markers in regorafenib treated cells. Importantly, *E-CAD* expression increased when cells were treated with 5 μM regorafenib while the expression of mesenchymal markers decreased upon regorafenib treatment except *VIM* (Figure 3A). There was a decrease in the actin stress fibers in regorafenib treated cells (Supplementary Figure 3). Moreover, regorafenib treatment also induced the expression of hepatic stem/progenitor markers *LGR5*, *AXIN2*, *CCND1*, *EpCAM* and CK19 (Figure 3B). Also, the expression of *ANXA3* which promotes angiogenesis, drug resistance, and stemness in HCC (Tong et al., 2015, 2018), and the expression of *KLF4* which is one of the Yamanaka factors that also regulates liver cancer stem cell plasticity (Karagonlar et al., 2020), increased upon regorafenib treatment (Figure 3B). EpCAM is a known Wnt/β-catenin signaling target gene and an important liver cancer stem cell marker (Yamashita et al., 2007; Terris et al., 2010). Regorafenib treatment increased membranous expression of EpCAM (Supplementary Figure 3). Strikingly, flow cytometry analysis showed that when cells were treated with Wnt3a/R-Spo1 in combination with regorafenib, the induction of EpCAM+ cell population was greatly enhanced (from 42.4 to 68.5%). On the other hand, CD133+ and CD24+ cell populations were reduced under combined treatment (Figure 3C). Importantly, when we knocked down β-catenin expression using a shβ-catenin plasmid (Figure 3D), the regorafenib induced increase in EpCAM was abolished. Although the mRNA expression of E-CAD also dropped, the protein level did not seem to be significantly altered (Figures 3E,F). Knock-down of β-catenin also affected the 3-D growth of cells and their colony forming ability. Moreover, knock-down of β-catenin rendered 3-D spheroids more sensitive to regorafenib (Figures 3G,H).

### TGF-β1 Treatment Decreases Regorafenib Induced E-CAD and p-β-Catenin Expression While Vimentin Expression Stays High

**FIGURE 4:**
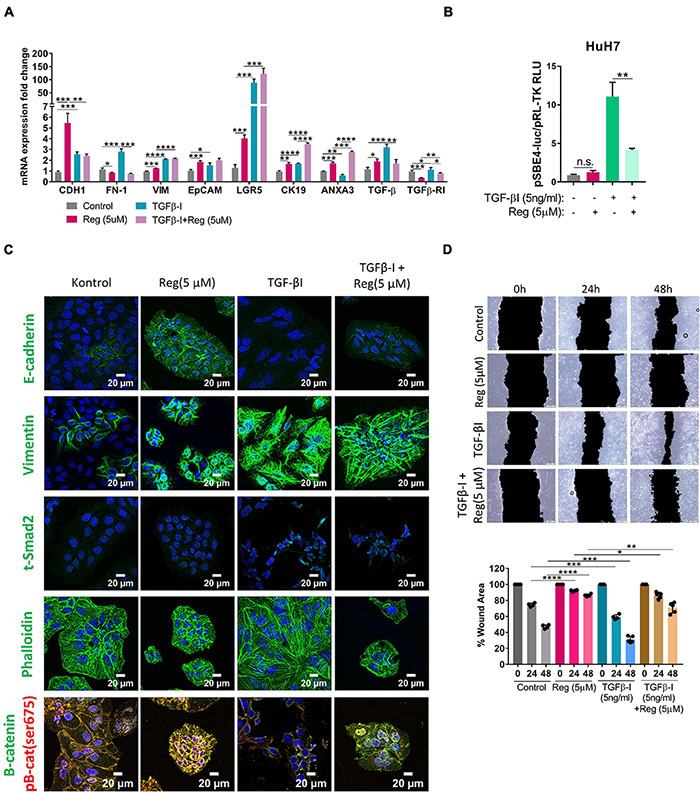
TGF-β treatment partially reverses regorafenib induced effects. **(A)** Expression of mesenchymal and epithelial genes were analyzed by q-PCR. Data represent the average of at least three independent experiments. **(B)** TGF-β reporter activity in basal and regorafenib treated cells was measured using pSBE4-Luc/pRL-TK construct. **(C)** Expressions of E-CAD, VIM, SMAD2 and β-catenin were analyzed in control and treated (TGF-β1, regorafenib, or combined) cells by immunofluorescence. Images were acquired on a Carl Zeiss LSM 880 AxioObserver confocal microscope with a C-Apochromat 40x/1.2 W, Korr FCS M27 objective. **(D)** Scratch assay was performed in control and treated (TGF-β1, regorafenib, or combined) cells. Wound area was calculated using ImageJ MRI wound healing tool. *p* > 0.05 (n.s.), *p* ≤ 0.05 (*), *p* ≤ 0.01 (**), *p* ≤ 0.001 (***), *p* ≤ 0.0001 (****). Error bars indicate standard deviation (SD).

EMT is known to be a critical step in acquisition of drug resistance (Aiello and Kang, 2019; Derynck and Weinberg, 2019) and TGF-β signaling is a master regulator of EMT (Reichl et al., 2012; Papageorgis, 2015; Hao et al., 2019). Interestingly, we detected an increase in the expression of TGF-β1 upon regorafenib treatment, although the expression of TGFβ-R1 was decreased (Figure 4A). When we treated cells with both TGF-β1 and regorafenib, the increase in the expression of E-CAD and EpCAM upon regorafenib treatment was partly inhibited (Figure 4A). On the contrary, regorafenib induced expression of LGR5 and CK19 was further augmented by TGF-β1 treatment (Figure 4A). To analyze the effect of regorafenib treatment on TGF-β signaling, we utilized a reporter plasmid. While TGFβ-1 treatment significantly increased TGF-β signaling, regorafenib treatment suppressed TGFβ-1 induced activation (Figure 4B).

Confocal staining also confirmed that while regorafenib treatment induced the membranous expression of *E-CAD*, and p-*β-CAT*, upon TGF-β1 treatment regorafenib induced increase in their expression was abolished (Figure 4C). Consistent with the RNA expression data, Vimentin expression was also significantly induced by Regorafenib treatment in these cells. Moreover, upon TGF-β1 treatment, we saw a further increase in Vimentin expression and an increase in actin stress fibers consistent with the acquisition of a more-mesenchymal morphology (Deguchi and Sato, 2009; Figure 4C). Consistently, while regorafenib treatment decreased cell motility, TGF-β1 treatment alone increased the motility of cells. When two treatments combined, the inhibitory effect of regorafenib on cell motility was partly attenuated by TGF-β1 (Figure 4D).

### Acquired Drug Resistance of HuH-7 Demonstrates Increased Mesenchymal Gene Expression and Augmented TGF-β Signaling

**FIGURE 5:**
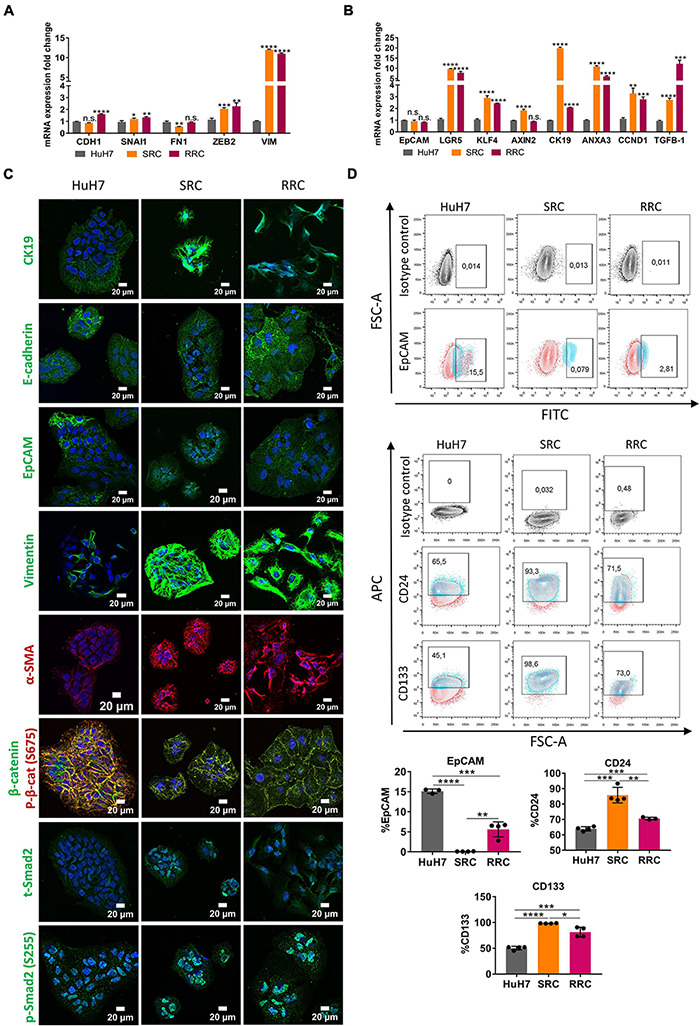
Regorafenib resistant cells have increased mesenchymal gene expression and cancer stem-cell marker expression. **(A)** Expression of mesenchymal and epithelial genes as well as **(B)** hepatic stem/progenitor genes were analyzed by q-PCR. Data represent the average of at least three independent experiments. *p* > 0.05 (n.s.), *p* ≤ 0.05 (*), *p* ≤ 0.01 (**), *p* ≤ 0.001 (***), *p* ≤ 0.0001 (****). Error bars indicate standard deviation (SD). **(C)** Expressions of CK19, E-CAD, EpCAM, VIM, a-SMA, SMAD2 and β-catenin were analyzed in parental and resistant cell lines by immunofluorescence. Images were acquired on a Carl Zeiss LSM 880 AxioObserver confocal microscope with a C-Apochromat 40x/1.2 W, Korr FCS M27 objective. **(D)** Cancer-stem cell marker (EpCAM, CD24 and CD133) positive cell populations were analyzed *via* BD LSR Fortessa flow cytometer in sorafenib resistant (SRC) and regorafenib resistant (RRC) cells.

**FIGURE 6:**
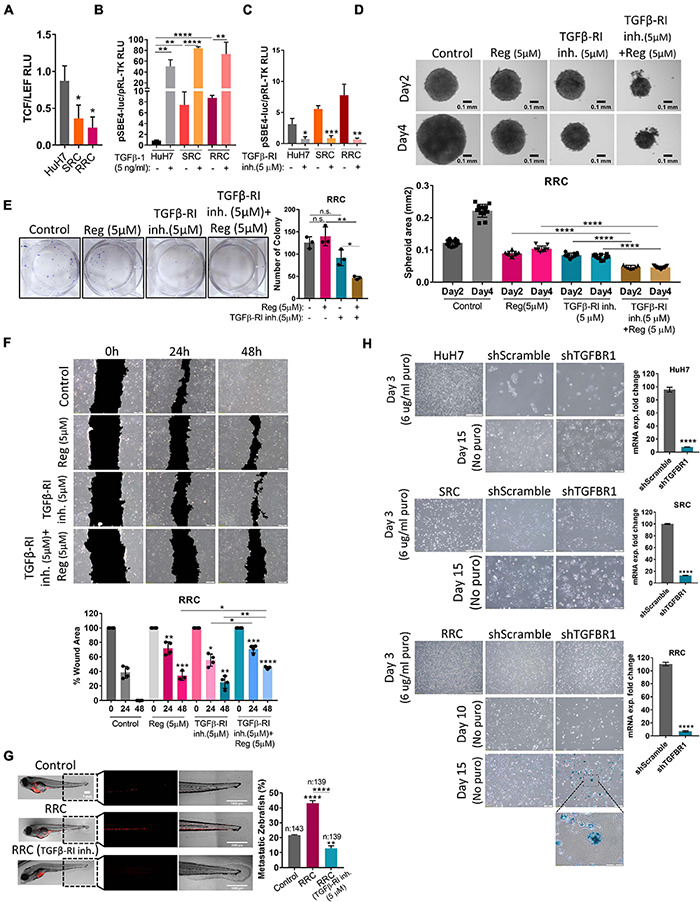
The augmented migration capacity of regorafenib resistant cells is mediated by the TGF-β pathway. **(A)** TCF/LEF reporter assay of resistant cells exhibit decreased TCF/LEF reporter activity. **(B)** TGF-β pathway activity was detected in HuH7, SRC and RRC cell lines after treatment with TGF-β1 or **(C)** TGFβ-RI inhibitor using pSBE4-Luc/pRL-TK reporter plasmid. **(D)** 3-D spheroids were formed from RRC cells with or without regorafenib and TGFβ-R1 treatment. Spheroid area was calculated using ImageJ. **(E)** Colony formation and **(F)** Scratch assay was performed in RRC cells with or without regorafenib and TGFβ-R1 treatment. Wound area was calculated using ImageJ MRI wound healing tool. **(G)** HuH7 cells, untreated RRC cells and TGFβ-R1 inhibitor treated RRC cells were implanted into the yolk sac of 2 dpf zebrafish embryos. 5 days after injection, migration to the tail was quantified. Representative images of zebrafish embryos were acquired on a Carl Zeiss LSM 880 AxioObserver confocal microscope with an EC Plan-Neofluar 10x/0.30 M27 objective. **(H)** SA-β-gal staining of control and TGFβ-R1 knocked down HuH7, SRC and RRC cells. *p* > 0.05 (n.s.), *p* ≤ 0.05 (*), *p* ≤ 0.01 (**), *p* ≤ 0.001 (***), *p* ≤ 0.0001 (****). Error bars indicate standard deviation (SD).

To analyze acquired drug resistance, we created Sorafenib and Regorafenib resistant cells by treating HuH7 cells with increasing doses of drugs starting with their IC50 values in long-term culture. Over 8–12 months, Sorafenib resistant (SRC) and Regorafenib resistant (RRC) cell lines were established and thereafter maintained constantly with media containing 7.2 μM Sorafenib (SRC line) or 8.4 μM Regorafenib (RRC line). MTT analysis confirmed that these resistant lines exhibited significantly higher IC50 values for both Sorafenib and Regorafenib, similar to mesenchymal-like HCC cell lines (Figure 1A and Supplementary Figure 3). Consistently, when compared to the parental HuH7 cell line, SRC and RRC cell lines had upregulation of several mesenchymal markers such as *SNAI1, ZEB2* and *VIM* and α-SMA (Figures 5A,C), supporting the acquisition of a mesenchymal phenotype. Interestingly, *CDH1* expression was also increased in the RRC line. Although *CDH1* is a well-defined epithelial marker, distant metastases of invasive cancers were shown to re-express *CDH1* which contributes to the establishment of metastatic foci. Moreover, similar to acute regorafenib treated cells, *LGR5*, *CK19*, *KLF4*, *ANXA3*, and *CCND1* gene expressions were also still significantly higher in SRC and RRC lines compared to parental cells (Figures 5B,C). Also, there was a significant upregulation in TGF-β1 expression in the SRC and RRC lines. Consistently, the expressions of total and p-SMAD2 (S255) were increased in SRC and RRC cell lines, while the expression of p-β-catenin was reduced (Figure 5C). Reporter assays also confirmed reduced β-catenin signaling and increased TGF-β signaling in the resistant cell lines (Figures 6A,B).

**FIGURE 7:**
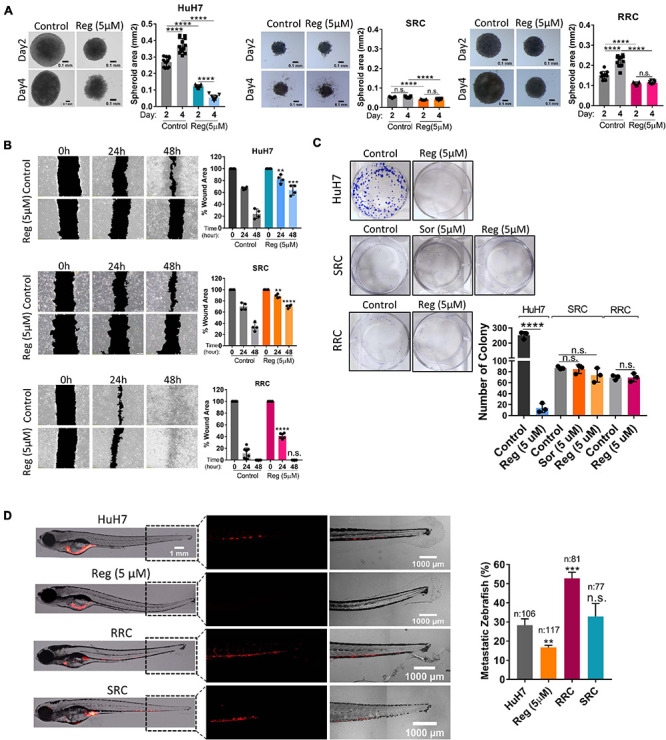
Regorafenib resistant cells demonstrate increased *in vitro* and *in vivo* migration ability. **(A)** 3-D spheroids were formed from parental HuH7, SRC and RRC cells with or without regorafenib treatment. Spheroid area was calculated using Image J. **(B)** Scratch assay and **(C)** Colony formation assay was performed in parental HuH7, SRC and RRC cells with or without regorafenib treatment. Wound area was calculated using Image J MRI wound healing tool. **(D)** Parental HuH7 cells, 5 μM regorafenib treated HuH7 cells, SRC and RRC lines were implanted into the yolk sac of 2 dpf zebrafish embryos. 5 days after injection, migration to the tail was quantified. Representative images of zebrafish embryos were acquired on a Carl Zeiss LSM 880 AxioObserver confocal microscope with a EC Plan-Neofluar 10x/0.30 M27 objective. *p* > 0.05 (n.s.), *p* ≤ 0.05 (*), *p* ≤ 0.01 (**), *p* ≤ 0.001 (***), *p* ≤ 0.0001 (****). Error bars indicate standard deviation (SD).

We also analyzed the expression of cancer stem cell surface markers *via* flow cytometry. We detected that EpCAM+ cell population decreased in both SRC and RRC lines. On the other hand, CD133 and CD24 expressing cell populations significantly increased in the resistant lines (Figure 5D). Moreover, although under 3-D growth conditions, resistant cell lines formed smaller spheroids, RRC spheroids were significantly more resistant to regorafenib treatment (Figure 7A). In addition, RRC cell line demonstrated significantly higher *in vitro* motility than parental and SRC cells in scratch assay (Figure 7B). Similarly, although basal colony forming capacity of SRC and RRC lines were lower than parental HuH7 cells, upon regorafenib treatment the colony forming ability of parental HuH7 cell line was greatly lost, while the colony forming abilities of SRC and RRC cells were not affected (Figure 7C).

### Acquired Drug Resistance Enhances the *in vivo* Migration Capacity of HuH7 Cells as Opposed to Acute Regorafenib Treatment in a Zebrafish Xenograft Model

To evaluate our *in vitro* results, we also tested the migration ability of SRC and RRC lines in a zebrafish xenograft model. 5 μM regorafenib treated HuH7 cells as well as SRC and RRC lines were implanted into the yolk sac of 2 dpf zebrafish embryos. 5 days after injection, migration to the tail was quantified. In total, 106 fishes from the control group, 117 fishes from the 5 μM regorafenib treated group, 77 fishes from the SRC group, and 81 fishes from the RRC group were counted. We detected migrated cells in 27% of zebrafish injected with HuH7 cells, while only 15 % of zebrafish injected with HuH7 cells treated with 5 μM regorafenib had migrated cells suggesting that regorafenib treated cells have reduced migration ability. Interestingly SRC line did not exhibit significantly higher migration ability (around 32% of zebrafish injected with SRC line had migration). However, we detected migrated cells around 53% of zebrafish injected with RRC line suggesting RRCs have significantly enhanced *in vivo* migration ability (Figure 7D).

### TGF-β RI Inhibition Significantly Prevents *in vitro* and *in vivo* Migration Ability of Regorafenib Resistant Cells

Wnt/β-catenin signaling increases upon acute regorafenib treatment. However, in SRC and RRC lines, we detected significantly reduced basal TCF/LEF activity (Figure 6A). Moreover Wnt3a/R-Spo-induced Wnt/β-catenin signaling was also diminished in SRC and RRC lines (Supplementary Figure 3). On the other hand, luciferase reporter assay using a reporter plasmid consisting of TGF-β responsive Smad-binding elements (pSBE4-Luc) demonstrated that basal TGF-β pathway activity is significantly higher in SRC (∼7.5-fold) and RRC (∼ 9-fold) lines compared to parental HuH7 cells (Figure 6B) consistent with significantly higher levels of TGF-β1 expression in these cells. We then utilized a TGFβ-R1 inhibitor that reduces TGF-β signaling in these cells (Figure 6C) and compared the various abilities of SRC and RRC cells with and without this inhibitor. Upon treatment of SRC and RRC spheroids with regorafenib and/or sorafenib and TGFβ-R1 inhibitor, cell death was augmented in the spheroids (Figure 6D and Supplementary Figure 4). Also compared to regorafenib alone, the combined treatment with TGFβ-R1 inhibitor and drugs significantly reduced colony formation (Figure 6E and Supplementary Figure 4) and *in vitro* migration of resistant cells (Figure 6F and Supplementary Figure 4). We also tested the effect of TGFβ-R1 inhibitor on the *in vivo* migration ability of RRC cells in the zebrafish model. In total, migration to the tail was counted in 143 fishes from the control group, in 139 fishes from the RRC group, and in 139 fishes from the RRC group treated with TGFβ-RI inhibitor (Figure 6G). We detected migrated cells in 21.6% of zebrafish injected with HuH7 cells, while 43% of zebrafish injected with the RRC group had migration again demonstrating the high migration ability of the RRC line. On the other hand, only 12.9% of zebrafish injected with the TGFβ-RI inhibitor treated RRCs had migrated suggesting that TGF-β pathway inhibition significantly prevents *in vivo* migration ability of regorafenib resistant cells.

**FIGURE 8:**
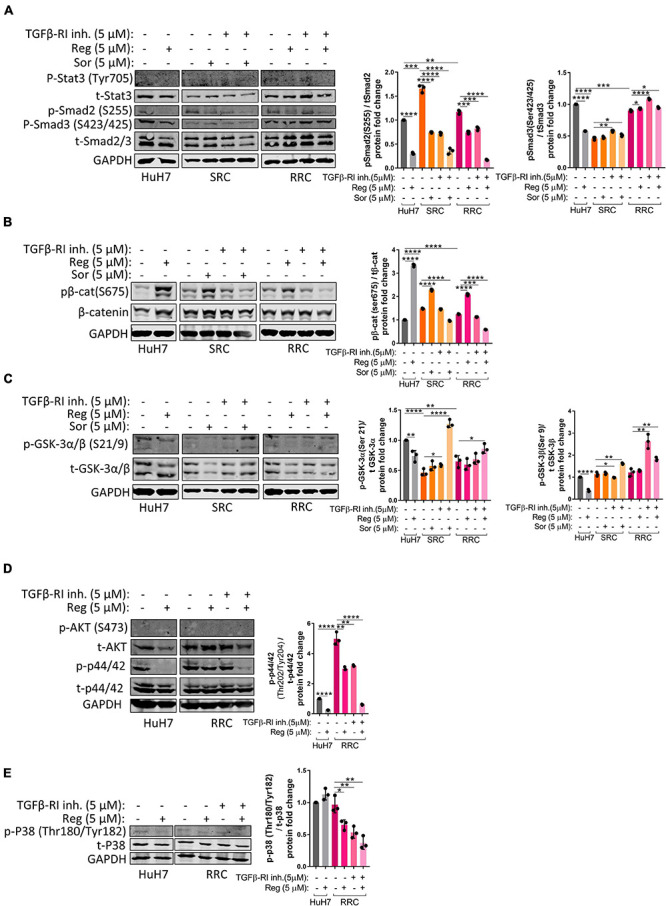
Western blot analysis of resistant cells. **(A–E)** Western blot analysis of untreated and treated (sorafenib, regorafenib, TGFβ-R1 or combined) parental HuH7, SRC and RRC cells. Bar graph represents the densitometric plot of protein expression in various groups. *p* > 0.05 (n.s.), *p* ≤ 0.05 (*), *p* ≤ 0.01 (**), *p* ≤ 0.001 (***), *p* ≤ 0.0001 (****). Error bars indicate standard deviation (SD).

Activation of the TGFβ pathway by TGF-β1 treatment is known to induce senescence in HCC cells (Senturk et al., 2010). In accordance with previous literature, we demonstrated that TGF-β1 treatment induces senescence in parental HuH7, SRC and RRC lines (Supplementary Figure 5). Interestingly, however, when we knocked down TGFβ-R1 in parental and resistant cells using a lentiviral plasmid, the majority of RRCs entered senescence while senescence was not detected in parental and SRC lines (Figure 6H). The induction of senescence by TGFβ-R1 knockdown in RRC line suggests that TGF-β pathway promotes growth and survival of regorafenib resistant cells. Phosphorylation of Smad2 at Ser255 *via* ERK was shown to serve as a STAT3 co-activator (Yoon et al., 2015). Western blot analysis demonstrated that in the RRC line, there are higher levels of phospho-STAT3, and phospho-SMAD2 (S255) (Figure 8). Importantly, regorafenib treatment reduced phosphorylations of SMAD2 and STAT3. However, this inhibition was even more significantly augmented when resistant cells were treated with regorafenib in combination with TGFβ-R1 inhibitor. Also, upon regorafenib treatment, phosphorylation of ERK1/2 was completely inhibited in HuH7 cells whereas in the resistant cell lines, the inhibition of pERK1/2 by regorafenib was not significant. However when regorafenib was combined with TGFβ-R1 inhibition, phosphorylation of ERK1/2 was completely inhibited even in SRC and RRC lines. On the other hand, upon regorafenib treatment phospho-β-catenin level increased in parental HuH7 and in the resistant lines. However when TGFβ-R1 inhibitor was applied in addition to regorafenib, the regorafenib induced increase in phospho-β-catenin was abolished while GSK3β phosphorylation increased (Figure 8).

## Discussion

Despite the recent advancements in HCC treatment, low response rates, drug toxicity, and treatment resistance usually followed by tumor relapse are still profound problems for HCC patients. The inter and intra-tumor heterogeneity of HCC plays a critical role in determining the patient’s therapy response. Thus, the future therapy direction of HCC should be focused on personalized treatment regimens and stratification of patients based on the efficacy of targeted therapies on HCC tumors with different molecular signatures (i.e., driver mutations, activated pathways) (Couri and Pillai, 2019; Pérez et al., 2020; Wang et al., 2020).

Human tumor-derived cell lines recapitulate HCC diversity and are widely used for studying mechanisms that drive drug resistance and sensitivity in HCC. Recent studies indicate that the various drug response rates among liver cancer cell lines were associated with the differences at transcriptomic level and the cell differentiation state. Among liver cancer cell lines, the most differentiated “hepatoblast-like” subgroup demonstrates the highest drug sensitivity (Caruso et al., 2019; Rebouissou and Nault, 2020). In our study, we also observed that hepatoblast-like HCC cell lines HuH7, HepG2, and Hep3B are more sensitive to regorafenib treatment compared to mesenchymal cell lines Snu387 and Snu449 (Figure 1A). However, we showed that although regorafenib induces cell death at lower doses in the “hepatoblast-like” cells, regorafenib treatment also activates Wnt/β-catenin signaling in these cells and induces enrichment of markers associated with hepatic stem/progenitor cells. Importantly, the activation of Wnt/β-catenin signaling can protect these cells from regorafenib induced apoptosis while the inhibition of Wnt/β-catenin signaling ameliorates cell death upon regorafenib treatment. Taken together, our results suggest that tumors with mutations that result in Wnt/β-catenin activation might have higher intrinsic regorafenib resistance. Importantly, a recent study by Harding et al. (2019) indicates that in HCCs patients treated with immune checkpoint inhibitors, the activating mutations of WNT/β-catenin signaling is associated with a decrease in drug response, shorter OS and innate resistance. Regorafenib is currently under clinical investigation as first-line therapy in combination with immunotherapy for advanced HCC (ClinicalTrials.gov. U.S. National Library of Medicine) and our data also supports the idea that stratification of patients based on the Wnt/β-catenin activation for high drug resistance could increase patient outcome in combinational therapies involving regorafenib.

On the other hand, upon long-term regorafenib treatment, Wnt signaling activity was diminished in HuH7 cells. Over 8–12 months, regorafenib treated HuH7 cells developed acquired regorafenib resistance and exhibited increased mesenchymal gene expression along with an induction of CD24 and CD133 cancer stem cell markers. Moreover, regorafenib resistant cells had enhanced TGF-β signaling activity and significantly higher migration capacity *in vivo* which can be reversed upon TGFβ-R1 inhibition. Thus, HuH7 cells, although they still express hepato-specific genes and fetal/progenitor markers, after long-time regorafenib treatment, also started to show properties of “mixed epithelial-mesenchymal” subgroup and the “mesenchymal-like” subgroup which are less differentiated and exhibit activation of the TGF-β pathway.

TGF-β exhibits multi-faceted roles and even opposite functions in distinct stages of cancer. During early stages of tumorigenesis TGF-β acts as a tumor suppressor, while in late stages it assumes an oncogenic role, promoting metastasis of tumor cells (Elliott and Blobe, 2005). Consistently, serum and tissue levels of TGF-β1 were found higher in metastatic HCC (Giannelli et al., 2002). In addition, high TGF-β1 levels correlate with tumor vascularity, metastasis and poor survival in HCC making TGF-β pathway a promising target in HCC therapy (Ito et al., 1995; Okumoto et al., 2004). However, although TGF-β pathway blockade has given promising results in preclinical models (Connolly et al., 2012; Katz et al., 2013), various anti-TGF-β agents that selectively and/or un-selectively inhibit TGF-β signaling tested in clinical trials, exhibited limited efficacies (Colak et al., 2017; Ciardiello et al., 2020; Nemunaitis et al., 2020). Due to its known roles in mediating inflammation, fibrogenesis, and immunomodulation in the tumor microenvironment, TGF-β inhibition is still a valuable target in combinational therapies. In a recent Phase 2 trial, Galunisertib, a small-molecule selective inhibitor of TGFβ-R1, was tested in combination with sorafenib in first-line patients with advanced HCC. The combination of two treatments showed a prolonged OS outcome compared to sorafenib alone (Kelley et al., 2019). The promising results of this study support further exploration of TGFβ-R1 inhibition in combination with other therapeutic agents. Importantly, a recent study indicated that HCC with active TGF-β signaling can be used as a potential biomarker for identifying the immune resistant tumors with poor prognosis (Chen et al., 2019). Immune-checkpoint inhibitors (ICIs) have been approved as second-line or first-line therapies for a list of malignancies including liver cancer (Gong et al., 2018; Havel et al., 2019). However, tumor response rates for these immune-checkpoint inhibitors are low, being less than 20% in HCC (Sangro et al., 2013; El-Khoueiry et al., 2017; Zhu et al., 2018). The highly immunosuppressive tumor environment in advanced HCC is believed to contribute to low treatment response of HCC to immune-checkpoint inhibitors (Prieto et al., 2015). Since TGF-β signaling has a role in immunomodulation of the tumor microenvironment, TGF-β blockade recently became the target of attention to enhance the ICI therapy especially for TGF-β-activated tumors. Several preclinical studies demonstrated that inhibition of TGF-β pathway could increase ICI drug response (Bai et al., 2019) and current ongoing clinical trials are exploring immunotherapeutic targeting of TGF-β signaling (Giannelli et al., 2011; Bai et al., 2019; Chen et al., 2019; Ungefroren, 2019; Groeneveldt et al., 2020; Li et al., 2020).

Our data by showing the role of TGF-β signaling in drug resistance of regorafenib resistant cells also merits the strategies involving TGF-β blockade in combination with regorafenib and immunotherapy in advanced HCC. TGF-β, as a potent regulator of tumor microenvironment, is a valuable candidate for therapies targeting the cross talk of cancer cells with the immune system and the stroma. Thus, despite the encouraging results, the clinical relevance of our study remains to be further established in preclinical animal models and in human patients.

## Data Availability Statement

The original contributions presented in the study are included in the article/Supplementary Material, further inquiries can be directed to the corresponding author/s.

## Ethics Statement

The animal study was reviewed and approved by Animal Experiments Local Ethics Committee of Izmir Biomedicine and Genome Center (IBG-AELEC).

## Author Contributions

MK, ZF, and EE: conceptualization, original draft preparation, and review and editing. MK and YA: methodology and experimentation. ZF, EE, GO, and SS: supervision. EE and GO: project administration and funding acquisition. All authors contributed to the article and approved the submitted version.

## Conflict of Interest

The authors declare that the research was conducted in the absence of any commercial or financial relationships that could be construed as a potential conflict of interest.

## Publisher’s Note

All claims expressed in this article are solely those of the authors and do not necessarily represent those of their affiliated organizations, or those of the publisher, the editors and the reviewers. Any product that may be evaluated in this article, or claim that may be made by its manufacturer, is not guaranteed or endorsed by the publisher.

## Supplementary Material

Supplementary Figure 1 Regorefenib treatment significantly inhibits **(A)** *in vitro* migration, **(B)** spheroid growth and **(C)** colony formation of hepatoblast-like cell lines.

Supplementary Figure 2 **(A)** Sub-G1 analysis of Wnt3/RSpo treated HuH7 cells **(B)** cleaved PARP analysis of Wnt3/RSpo treated HepG2 cells. **(C)** cleaved PARP analysis of IWR-1 treated cells Hep3B and HepG2 cells.

Supplementary Figure 3 **(A)** Phase-contrast image, **(B)** phalloidin staining and **(C)** EpCAM staining of acute regorafenib treated cells. **(D)** Phase contrast images and **(E)** TCF/LEF activity of resistant cell lines.

Supplementary Figure 4 **(A)***In vitro* migration, **(B)** spheroid formation and **(C)** colony formation of TGFβ-R1 treated SRC cells.

Supplementary Figure 5 **(A)** SA-β-gal staining of TGF-β1 treated, TGF β-R1 treated and DOX treated cells. **(B)** Spheroid formation of sh-TGF β-R1 cells.

Supplementary Table 1 qPCR Taqman specific primers.
