# Supplementary figures and images for: Changes in Wnt and TGF-β Signaling Mediate the Development of Regorafenib Resistance in Hepatocellular Carcinoma Cell Line HuH7
Source: Front Cell Dev Biol. 2021 Aug 11;9:639779. doi: 10.3389/fcell.2021.639779 (PMC8386122; doi:10.3389/fcell.2021.639779)

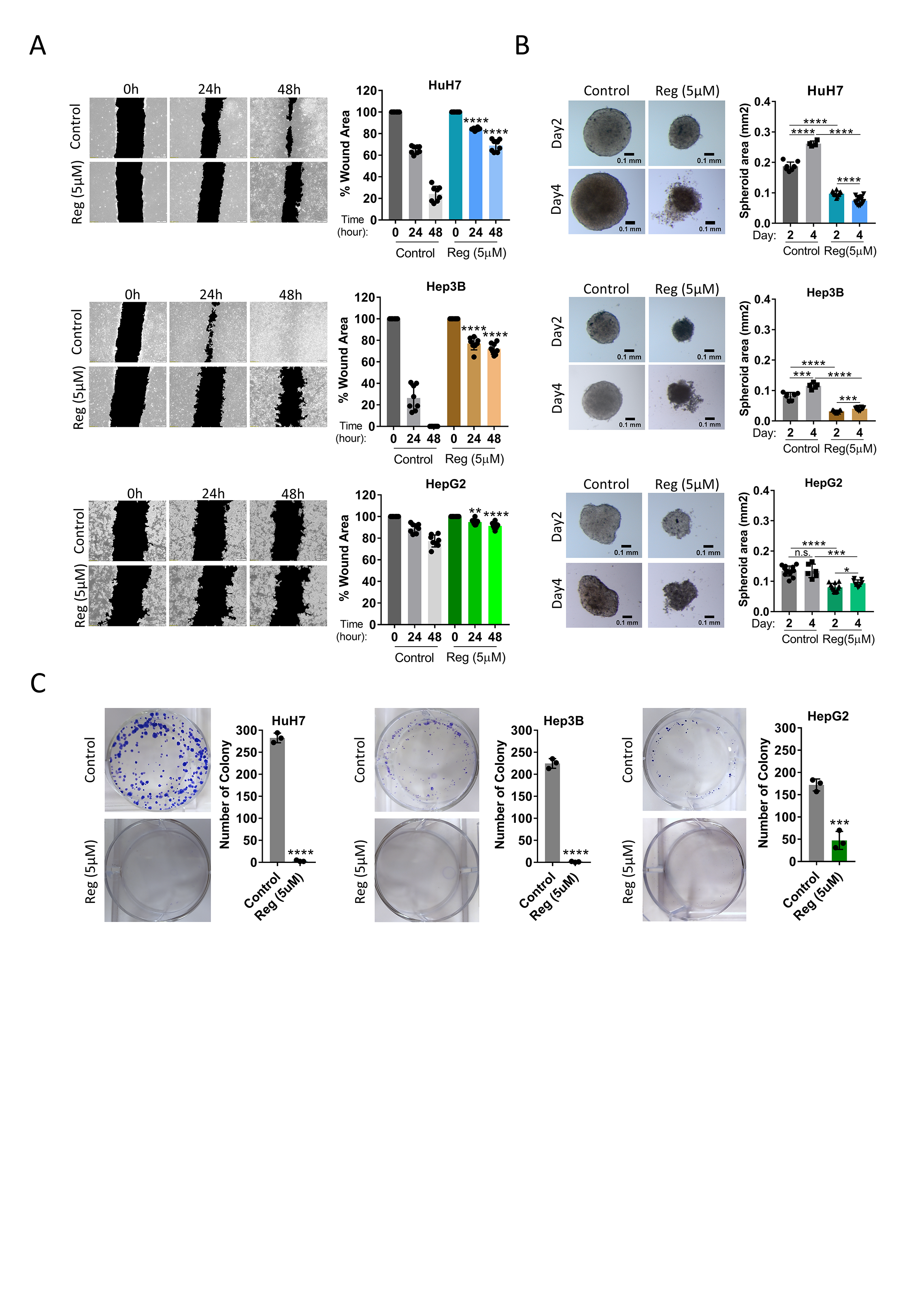

Supplement: Supplementary Figure 1 — Regorefenib treatment significantly inhibits (A) in vitro migration, (B) spheroid growth and (C) colony formation of hepatoblast-like cell lines. [file Image_1.TIF]

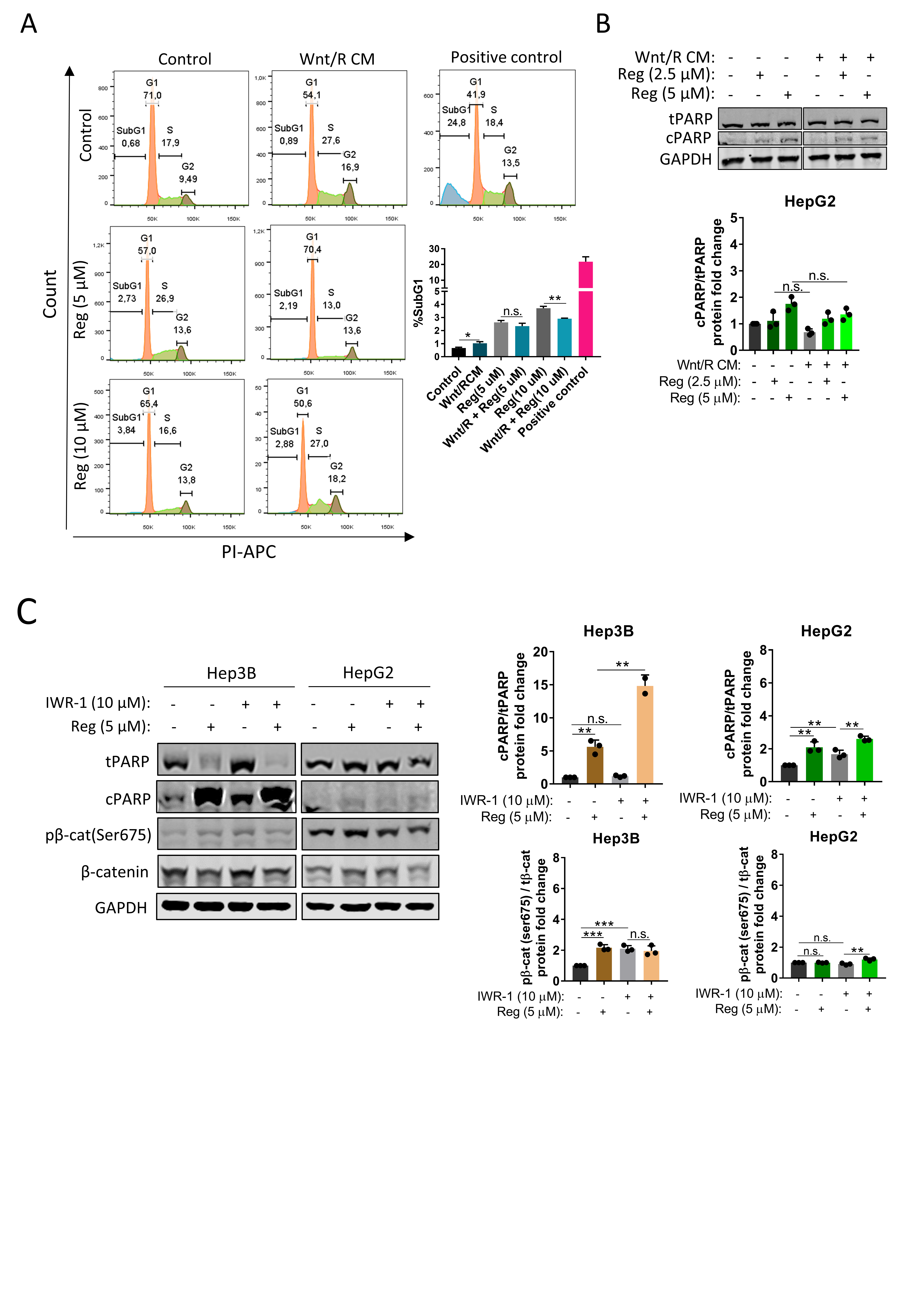

Supplement: Supplementary Figure 2 — (A) Sub-G1 analysis of Wnt3/RSpo treated HuH7 cells (B) cleaved PARP analysis of Wnt3/RSpo treated HepG2 cells. (C) cleaved PARP analysis of IWR-1 treated cells Hep3B and HepG2 cells. [file Image_2.TIF]

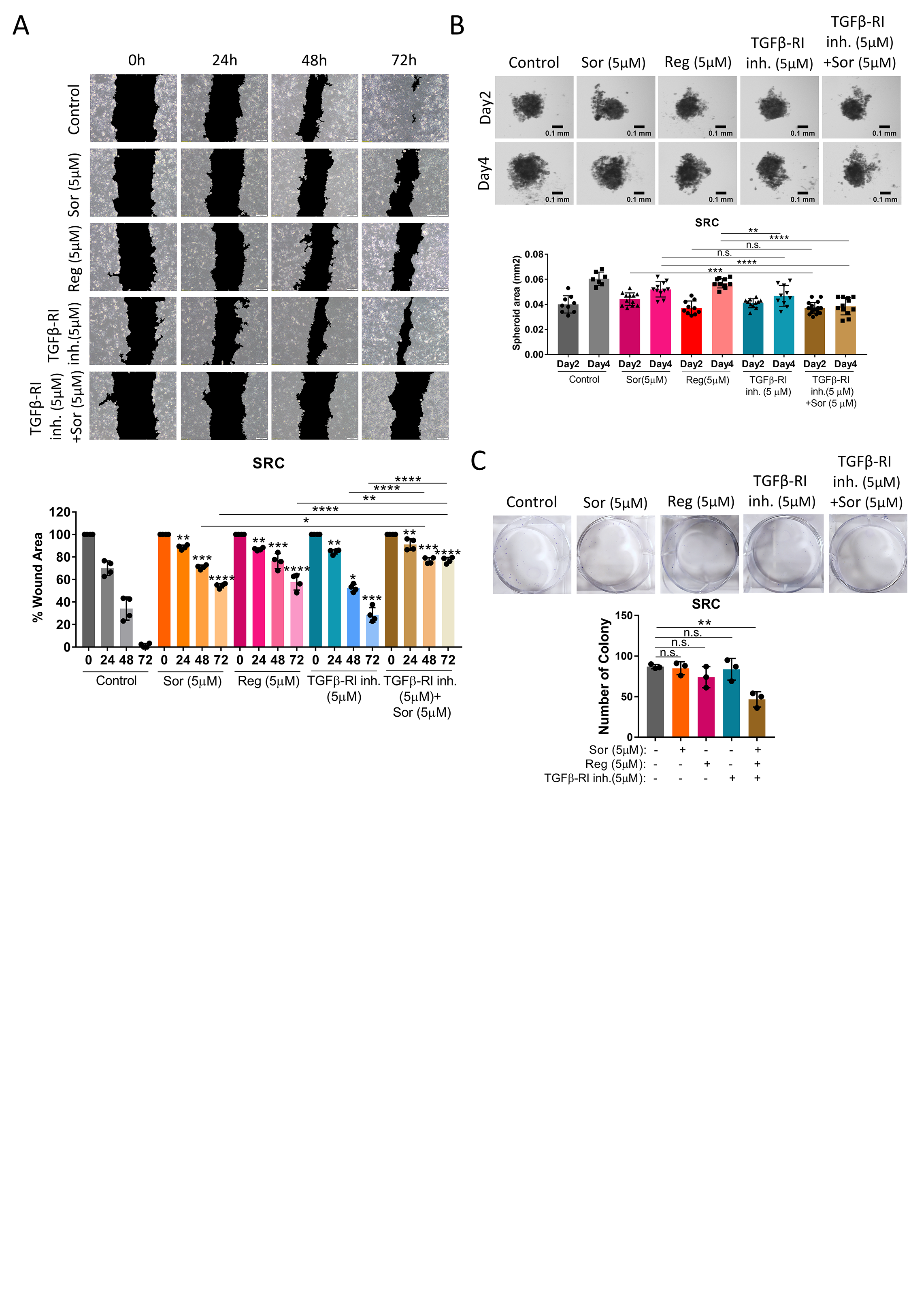

Supplement: Supplementary Figure 3 — (A) Phase-contrast image, (B) phalloidin staining and (C) EpCAM staining of acute regorafenib treated cells. (D) Phase contrast images and (E) TCF/LEF activity of resistant cell lines. [file Image_3.TIF]

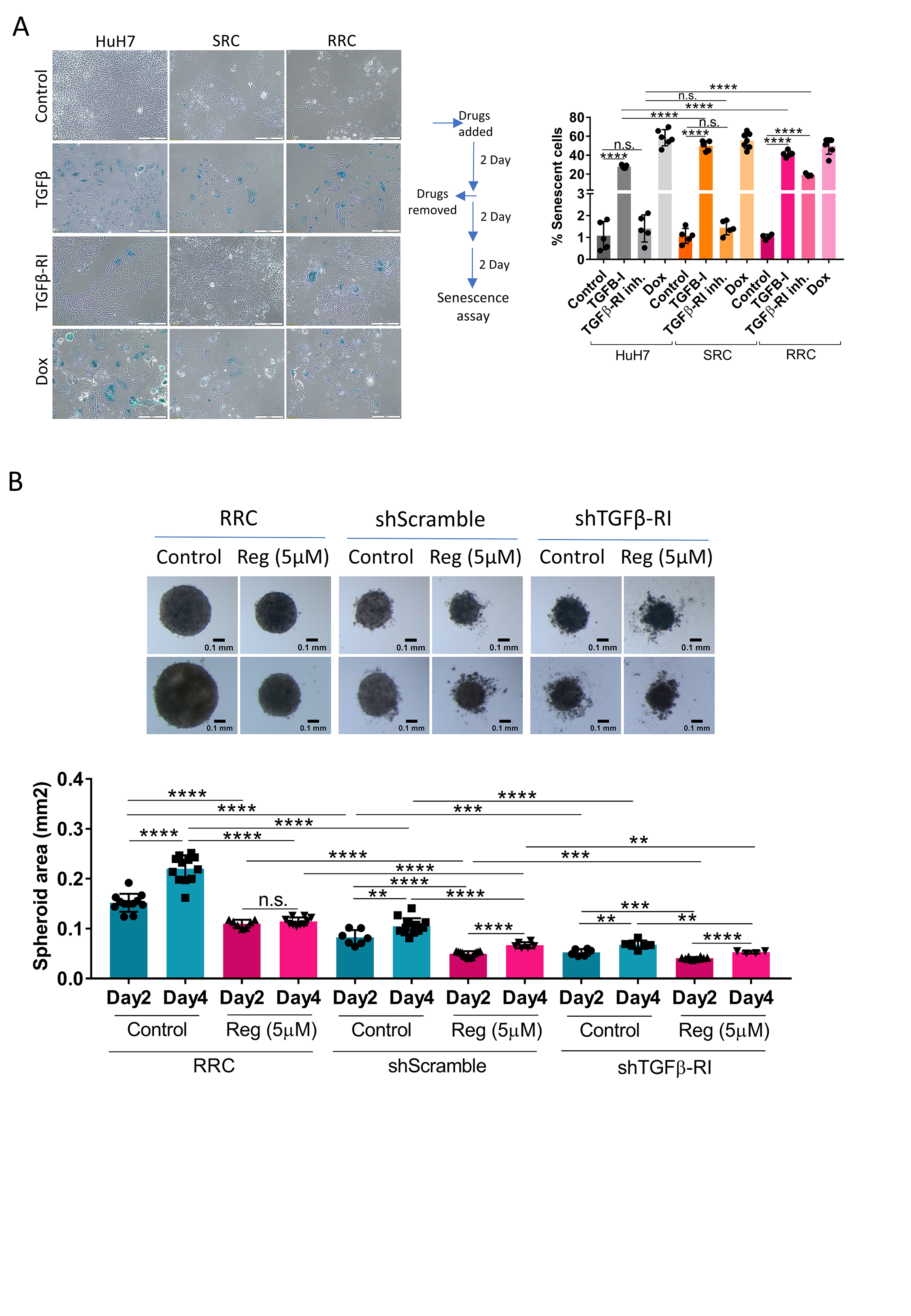

Supplement: Supplementary Figure 4 — (A)In vitro migration, (B) spheroid formation and (C) colony formation of TGFβ-R1 treated SRC cells. [file Image_4.TIF]

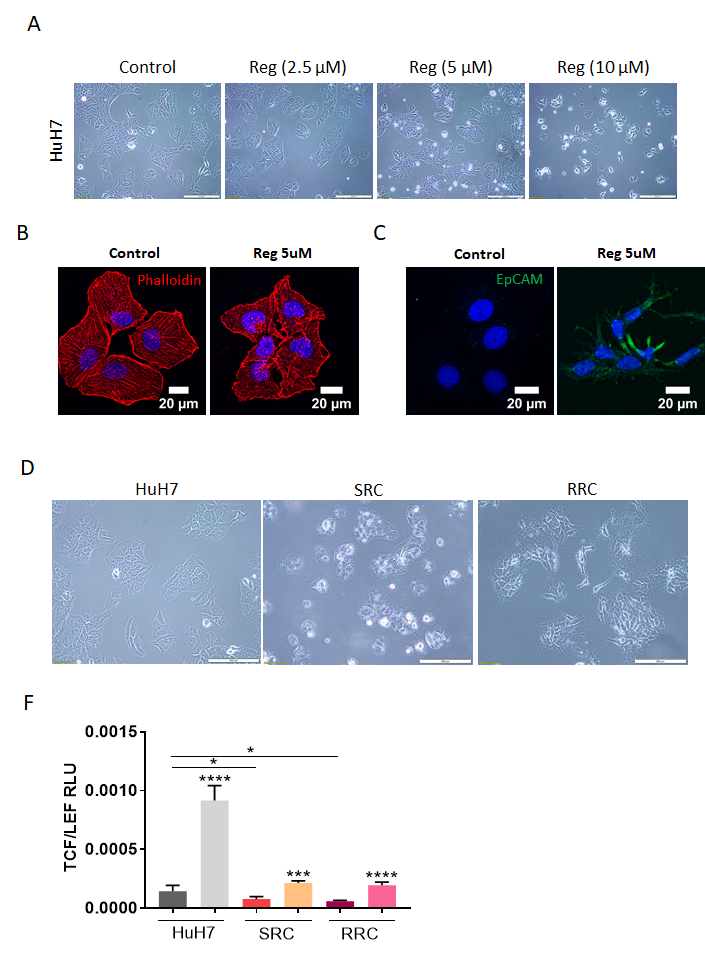

Supplement: Supplementary Figure 5 — (A) SA-β-gal staining of TGF-β1 treated, TGF β-R1 treated and DOX treated cells. (B) Spheroid formation of sh-TGF β-R1 cells. [file Image_5.TIF]
