# Supplementary material for: Changes in Wnt and TGF-β Signaling Mediate the Development of Regorafenib Resistance in Hepatocellular Carcinoma Cell Line HuH7
Source: Front Cell Dev Biol. 2021 Aug 11;9:639779. doi: 10.3389/fcell.2021.639779 (PMC8386122; doi:10.3389/fcell.2021.639779)
Supplement: Supplementary Table 1 — qPCR Taqman specific primers. [file Table_1.docx]

| **Gene ID** | **Forward primer** | **Reverse primer** | **Prop no** |
| --- | --- | --- | --- |
| **ANXA3** | **cagaaatatcagccaaaaggacat** | **ggcgtgttcctcacacaat** | **56** |
| **AXIN2** | **gatatccagtgatgcgctga** | **actgcccacacgataaggag** | **56** |
| **CCND1** | **cggactacaggggagttttg** | **ctctgctgctcgctgctac** | **1** |
| **CDH1** | **accccctgttggtgtcttta** | **tgtatgtggcaatgcgttct** | **41** |
| **CK19** | **agtaccagcggctcatgg** | **cttcctgtccctcgagca** | **15** |
| **EpCAM** | **ccatgtgctggtgtgtgaa** | **tgtgttttagttcaatgatgatcca** | **3** |
| **FN1** | **gacgcatcacttgcacttct** | **gcaggtttcctcgattatcct** | **1** |
| **KLF4** | **cgttccagtgccaaaaatg** | **catgtgtaaggcgaggtggt** | **85** |
| **LGR5** | **accagactatgcctttggaaac** | **tcccagggagtggattctatt** | **78** |
| **RPL41** | **ggccttagcgccattttt** | **ttggacctctgcctcatctt** | **41** |
| **SNAI1** | **gagctgcaggactctaatcca** | **cggtggggttgaggatct** | **10** |
| **TGFB** | **ggagcggaggaaggagtc** | **ctcttctcccgaccagctc** | **63** |
| **TGFB-R1** | **gcagacttaggactggcagtaag** | **agaacttcaggggccatgt** | **5** |
| **VIM** | **agatggcccttgacattgag** | **cagggaggaaaagtttggaa** | **11** |
| **ZEB2** | **ttgaagtgatgcatgtgtgaat** | **accaccttacaaaggctttcttt** | **85** |
